# Stationary tissue background correction increases the precision of clinical evaluation of intra-cardiac shunts by cardiovascular magnetic resonance

**DOI:** 10.1038/s41598-020-61812-7

**Published:** 2020-03-19

**Authors:** Jannike Nickander, Magnus Lundin, Goran Abdula, Jonas Jenner, Eva Maret, Peder Sörensson, Einar Heiberg, Andreas Sigfridsson, Martin Ugander

**Affiliations:** 1 Department of Clinical Physiology, Karolinska University Hospital, Karolinska Institutet, Stockholm, Sweden; 2 Department of Medicine, Unit of Cardiology, Karolinska Institutet, and Karolinska University Hospital, Stockholm, Sweden; 3 Department of Clinical Physiology, Lund University, and Skåne University Hospital, Lund, Sweden; 4 Wallenberg Centre for Molecular Medicine, Lund University, Skåne University Hospital, Lund, Sweden; 5 Kolling Institute, Royal North Shore Hospital, and Northern Clinical School, Sydney Medical School, University of Sydney, Sydney, Australia

**Keywords:** Cardiovascular diseases, Magnetic resonance imaging

## Abstract

We aimed to evaluate the clinical utility of stationary tissue background phase correction for affecting precision in the measurement of Qp/Qs by cardiovascular magnetic resonance (CMR). We enrolled consecutive patients (*n* = 91) referred for CMR at 1.5T without suspicion of cardiac shunt, and patients (*n* = 10) with verified cardiac shunts in this retrospective study. All patients underwent phase contrast flow quantification in the ascending aorta and pulmonary trunk. Flow was quantified using two semi-automatic software platforms (SyngoVia VA30, Vendor 1; Segment 2.0R4534, Vendor 2). Measurements were performed both uncorrected and corrected for linear (Vendor 1 and Vendor 2) or quadratic (Vendor 2) background phase. The proportion of patients outside the normal range of Qp/Qs was compared using the McNemar’s test. Compared to uncorrected measurements, there were fewer patients with a Qp/Qs outside the normal range following linear correction using Vendor 1 (10% vs 18%, *p* < 0.001), and Vendor 2 (10% vs 18%, *p* < 0.001), and following quadratic correction using Vendor 2 (7% vs 18%, *p* < 0.001). No patient with known shunt was reclassified as normal following stationary background correction. Therefore, we conclude that stationary tissue background correction reduces the number of patients with a Qp/Qs ratio outside the normal range in a consecutive clinical population, while simultaneously not reclassifying any patient with known cardiac shunts as having a normal Qp/Qs. Stationary tissue background correction may be used in clinical patients to increase diagnostic precision.

## Introduction

Blood flow quantification is an important part of clinical cardiovascular hemodynamic assessment using cardiovascular magnetic resonance (CMR). Phase contrast velocity encoded CMR (PC-CMR) is reproducible^1^, used for quantitative assessment in valvular disease^2–5^, and used to quantify the ratio of flow between the pulmonary and systemic circulation (Qp/Qs) in order to detect and quantify cardiac shunts^6^. However, both the magnitude and precision of Qp/Qs are influenced by measurement errors in the flow quantifications in the respective vessel. One source of error is eddy current effects due to field inhomogeneity, which introduce phase distortions or spatially dependent phase offsets^7,8^. Therefore, post-processing correction algorithms have been proposed to reduce measurements errors, and these include linear^9^ and quadratic stationary tissue background phase correction^10^.

The linear correction method assumes that the phase offset errors are spatially dependent in a linear fashion. The method fits a flat surface via the time average of stationary pixels in the velocity-encoded phase images, and these values are then subtracted from the velocity-encoded images. The quadratic correction method performs the fitting of the stationary pixels with a second-degree polynomial assumption. The proposed methods have not yet been evaluated systematically in a larger clinical population without congenital heart disease, and the effect on precision and magnitude has not been reported. Several clinical software products are in use and have been evaluated head-to-head in phantoms^11^. However, it is currently unknown if these software products differ in performance in a patient population. Furthermore, a number of patient characteristics including sex, age, body surface area (BSA), and image angulation might influence the stationary tissue background correction. Therefore, these characteristics were investigated for changes in precision and magnitude of Qp/Qs following correction. We hypothesized that the precision in Qp/Qs in a clinical population without known cardiac shunts, would increase following stationary tissue background correction.

## Methods

### Study population

Consecutive patients (*n* = 91, 62% male, age (median [interquartile range]) 52 [39–62] years) referred for clinical CMR, examined between January and September 2014, were retrospectively enrolled. As the variability of Qp/Qs is not known, a power calculation was not possible, thus fixed time points for inclusion were predetermined. Patients were eligible for the study if: the clinical report had no mention of cardiac shunts or malformations of the great vessels, the atria were of normal size, the ratio of left to right ventricular end diastolic diameters was <1, no persistent arrhythmias defined as a standard deviation of the mean R-R interval during the phase contrast acquisitions exceeding 10% of the R-R interval for either acquisition were present, and there were no extensive fold-over artifacts. Furthermore, consecutive patients (*n* = 10, 30% male, age (mean ± SD 44 ± 14 years) with known cardiac shunt by an independent method (echocardiography, computed tomography or invasive procedure) imaged during the same time period were included.

### Image acquisition

CMR was conducted in the supine position using a 1.5T scanner (Siemens Aera, Erlangen, Germany) with 34 surface coil elements (spine and body matrix coils). A clinically available phase contrast flow quantification sequence with retrospective electrocardiographic (ECG) gating was used to acquire through-plane phase contrast images of the proximal ascending aorta and the proximal main pulmonary artery, as recommended in guidelines^12^. Typical imaging parameters included: field of view of 286 × 340 mm^2^, matrix 256 × 216, slice thickness 5 mm, repetition time (TR) 5.13 ms, echo time (TE) 2.83 ms, flip angle 20°, bandwidth 455 Hz, and velocity encoding (VENC) 100–200 cm/s, adapted to avoid aliasing. The acquisition duration was set to 117 heart beats and was performed during free breathing with three-fold averaging to suppress respiratory motion artifacts.

A balanced steady-state free precession cine sequence, with retrospective ECG gating, covering the left ventricle was used for assessing left ventricular function. Typical imaging parameters included: flip angle = 68°, voxel size = 2.0 × 2.0 × 8.0 mm^3^, TR/TE = 2.85/1.19 ms, matrix size = 143 × 256, and field of view 303 × 360 mm^2^.

### Image analysis

**Figure 1:**
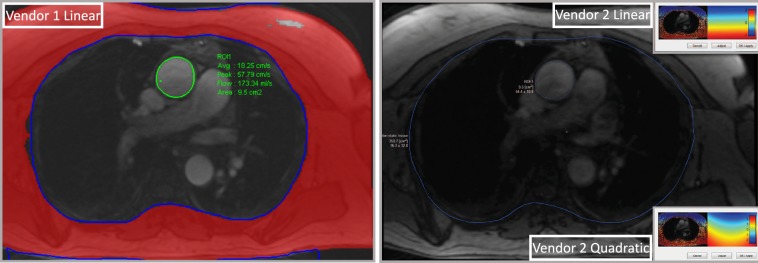
Stationary tissue background correction in Vendor 1 and Vendor 2. Non-static tissue was excluded by regions of interest in blue. The amount of static tissue was maximized by increasing the phase deviation threshold, which is shown in Vendor 1 (to the left) in red. In Vendor 2, linear correction is shown in the smaller window in the top right corner, and quadratic correction is shown in the smaller window in the bottom right corner.

Flow quantification in the ascending aorta and main pulmonary artery was performed by semi-automated methods with manual adjustments by one observer using dedicated software by Vendor 1 (SyngoVia Software, VA30, Siemens, Erlangen, Germany), and a freely available software by Vendor 2 (Segment version 2.0 R4534, Medviso AB, Lund, Sweden)^13^, respectively. Regions of interest (ROI) in the ascending aorta and main pulmonary artery were automatically delineated. Flow velocity measurements were performed both uncorrected and with linear background correction using Vendor 1, and both uncorrected and with linear and quadratic background correction using Vendor 2, respectively. Background correction was performed by first maximizing the amount of static tissue included in the calculation by increasing the phase deviation threshold and then drawing an excluding ROI over non-static tissue, such as lungs, and major vessels, Fig. 1. The normal range of Qp/Qs was defined as 0.9 to 1.2^14,15^.

To determine the intra-observer and inter-observer variability, 20 patients were reevaluated for uncorrected and corrected flow measurements.

Quantification of left ventricular volume, ejection fraction and myocardial mass was performed with Vendor 1 by carefully outlining the epi- and endocardial borders in end diastole and end systole in the cine short-axis stack. Atrial area was measured by carefully outlining the atria in end systole, and ventricular end diastolic diameter by measuring diameter in end diastole in a four chamber cine view. BSA was calculated by the DuBois and DuBois formula^16^. Volumetric measurements and myocardial mass were indexed to BSA.

### Statistical analysis

Continuous, normally distributed variables as determined by the Kolmogorov-Smirnov test were reported as mean ± standard deviation. Continuous, non-normally distributed variables, were reported as median [interquartile range]. Categorical variables were reported as percentages, and evaluated for differences with McNemar’s test. Statistical analysis was performed using Microsoft Excel (Microsoft, Redmond, Washington, USA) and IBM SPSS Statistics for Macintosh, version 23 (IBM Corp, Armonk, N.Y, USA). Intra-observer and inter-observer variability are presented as the intra-class correlation coefficient (ICC). Patient characteristics were dichotomized according to high and low values in relation to the median, and assessed for changes in precision following background correction. Change in precision was defined as change in measurement variability determined by the F-test in normally distributed data, and by non-parametric Levene’s test in non-normally distributed data. Normally distributed data were compared by the paired or unpaired t-test as appropriate. Non-normally distributed data were compared using the Mann-Whitney U-test or Wilcoxon signed rank test as appropriate. Statistical significance was defined as *p* < 0.05. However, Bonferroni correction was applied for analyses of multiple patient characteristics, where *m* = 20 and *alpha* = 0.05, resulting in *p* < 0.0025 as the level of statistical significance.

### Ethical approval

The study was approved by the Regional Ethics Review Board in Stockholm, ID nr: 2011/1077-31/3, and all patients provided written informed consent. All study procedures were carried out in accordance with Good Clinical Practice (GCP).

### Study subjects overlap

Eighteen study subjects have been studied in a previous study unrelated to flow quantification (Nickander J. *et al*. J Cardiovasc Magn Reson. 2017;19(1):41).

## Results

### Study population

Baseline characteristics of the clinical population are summarized in Table 1, and in Table 2 for the patients with known shunts.

**Table 1:** Baseline characteristics for the study population.

Characteristics	All (*n* = 91)
**CMR Diagnosis**, ***n*** **(%)**	
Normal Myocarditis Myocardial infarction Heart failure Hypertrophic cardiomyopathy Valvular disease Hypertrophic right heart Takotsubo cardiomyopathy Non-compaction cardiomyopathy Diffuse myocardial fibrosis Cardiac amyloidosis	31 (34) 16 (18) 13 (14) 13 (14) 4 (4) 4 (4) 1 (1) 5 (5) 1 (1) 1 (1) 2 (2)
Age, years	50 ± 16
Weight, kg	79 ± 18
Height, cm	174 ± 9
BSA, m^2^	1.9 ± 0.3
LA area, cm^2^	24 ± 5
RA area, cm^2^	21 ± 5
LV EDD, mm	53 ± 8
RV EDD, mm	44 ± 7
LVEDV, ml	183 ± 71
LVEDVI, ml/m^2^	95 ± 36
LVESV, ml	90 ± 68
LVESVI, ml/m^2^	47 ± 36
LVSV, ml	93 ± 22
LVSVI, ml/m^2^	48 ± 10
LVEF, %	54 ± 12
LVM, g	148 ± 48
LVMI, g/m^2^	76 ± 21

Data presented as mean ± standard deviation. BSA = body surface area, LA = left atrium; LVEDD = left ventricular end diastolic diameter, LVEDV = left ventricle end diastolic volume, LVESV = left ventricle end systolic volume, LVSV = left ventricle stroke volume, LVEF = left ventricle ejection fraction, LVM = left ventricular mass, RA = right atrium, RVEDD = right ventricular end diastolic diameter. An ‘I’ indicates volumes and mass indexed to BSA.

**Table 2:** Baseline characteristics for patients with known shunts.

Characteristics	All (*n* = 10)
**Shunt diagnosis**, ***n***	
Ventricular septal defect Anomalous origin of the PA Atrial septal defect Patent ductus arteriosus Transposition of the great arteries Aortopulmonary window Communication between descending aorta and the lung	3 2 1 1 1 1 1
Age, years	44 ± 14
Weight, kg	73 ± 17
Height, cm	172 ± 10
BSA, m^2^	1.9 ± 0.3
LA area, cm^2^	29 ± 16
RA area, cm^2^	27 ± 15
LV EDD, mm	52 ± 18
RV EDD, mm	46 ± 9
LVEDV, ml	189 ± 38
LVEDVI, ml/m^2^	91 ± 37
LVESV, ml	84 ± 30
LVESVI, ml/m^2^	40 ± 17
LVSV, ml	104 ± 24
LVSVI, ml/m^2^	57 ± 15
LVEF, %	56 ± 10
LVM, g	109 ± 42
LVMI, g/m^2^	63 ± 13

Data presented as mean ± standard deviation. BSA = body surface area, LA = left atrium; LVEDD = left ventricular end diastolic diameter, LVEDV = left ventricle end diastolic volume, LVESV = left ventricle end systolic volume, LVSV = left ventricle stroke volume, LVEF = left ventricle ejection fraction, LVM = left ventricular mass, RA = right atrium, RVEDD = right ventricular end diastolic diameter. An ‘I’ indicates volumes and mass indexed to BSA.

### Normalization of Qp/Qs

**Figure 2:**
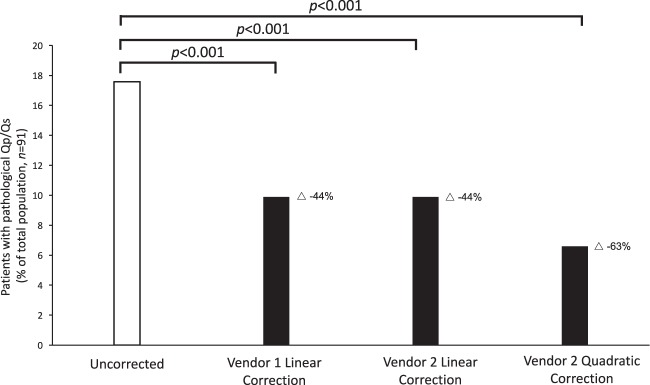
Reduction of the number of patients with pathological Qp/Qs following stationary tissue background correction (*n* = 91). Stationary tissue background correction reduces the number of patients with pathological Qp/Qs. Triangle denotes reduction in patients with pathological Qp/Qs in percent. *P*-values denote McNemar’s test.

Using Vendor 1, sixteen of the ninety-one patients (18%) had a pathological Qp/Qs before correction, and nine (10%) following correction. Nine of sixteen patients (56%) were normalized following linear correction. Using Vendor 2, sixteen patients (18%) also had a pathological Qp/Qs before correction and nine patients (10%) following correction. Seven of sixteen patients (44%) were normalized following linear correction. Using Vendor 2 and quadratic correction, sixteen patients (18%) had a pathological Qp/Qs before correction and six patients (7%) following correction. Out of the sixteen with a pathological Qp/Qs prior to correction, twelve were normalized following correction (75%), Fig. 2.

### Qp/Qs in patients with known shunts

**Figure 3:**
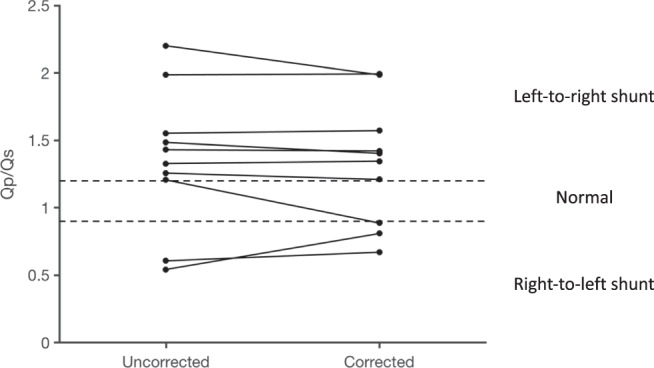
Stationary tissue background correction in patients with a verified shunt. The figure shows Qp/Qs before and following linear stationary background correction using Vendor 1. No patient was reclassified as normal following correction. The patient that is classified as a left-to-right shunt prior to correction and a right-to-left shunt following correction had a complex heart condition including a communication between the descending aorta and the lung, and pulmonary regurgitation. The pulmonary backward volume was not quantifiable before background correction, why this patient is correctly classified a right-to-left shunt following background correction.

No patient with a known shunt was reclassified as being normal following stationary background correction, Fig. 3. One patient had a complex heart condition including a communication between the descending aorta and the lung, and pulmonary regurgitation. The pulmonary backward volume was not quantifiable before background correction, why this patient was classified as a left-to-right shunt prior to correction and a right-to-left shunt following correction.

### Intra-observer and inter-observer variability

Intra-observer and inter-observer variability was low, see Table 3.

**Table 3:** Intra- and inter-observer variability.

Vendor 1	Intra-class correlation	
	Qp/Qs uncorrected	Qp/Qs corrected
Intra-observer	0.97, *p* < 0.001	0.98, *p* < 0.001
Inter-observer	0.98, *p* < 0.001	0.96, *p* < 0.001
Vendor 2, linear	**Qp/Qs uncorrected**	**Qp/Qs corrected**
Intra-observer	0.95, *p* < 0.001	0.90, *p* < 0.001
Inter-observer	0.95, *p* < 0.001	0.90, *p* < 0.001
Vendor 2, quadratic	**Qp/Qs uncorrected**	**Qp/Qs corrected**
Intra-observer	0.95, *p* < 0.001	0.70, *p* < 0.01
Inter-observer	0.95, *p* < 0.001	0.73, *p* < 0.01

### Phase contrast vs cine

Data on aortic flow using phase contrast compared to stroke volume from cine are summarized in Table 4.

**Table 4:** Inter-method comparison of aortic flow.

Vendor 1	Inter-method comparison	
	Phase contrast aortic flow (ml)	Stroke volume by cine (ml)
Uncorrected	74 ± 20	93 ± 22, *p* < 0.001
Corrected	77 ± 20	93 ± 22, *p* < 0.001
Vendor 2, linear	**Phase contrast aortic flow (ml)**	**Stroke volume by cine (ml)**
Uncorrected	75 ± 20	93 ± 22, *p* < 0.001
Corrected	81 ± 20	93 ± 22, *p* < 0.001
Vendor 2, quadratic	**Phase contrast aortic flow (ml)**	**Stroke volume by cine (ml)**
Uncorrected	75 ± 20	93 ± 22, *p* < 0.001
Corrected	81 ± 20	93 ± 22, *p* < 0.001

*P*-values denote paired t-test.

### Patient characteristics, Qp/Qs precision and vendor comparison

No patient characteristics affected precision in Qp/Qs. Precision in Qp/Qs was increased by background correction using Vendor 1, and quadratic but not linear correction using Vendor 2. There was no difference in corrected Qp/Qs between the Vendors. These results are summarized in the Supplemental Material.

## Discussion

The main finding of the study is that stationary tissue background correction increases precision in a clinical population by reducing the number of clinical patients with a Qp/Qs outside the normal range. Furthermore, stationary tissue background correction does not reclassify patients with a known cardiac shunt, by an independent method, to normal. This illustrates the ability of stationary tissue background correction to increase diagnostic precision in clinical flow quantification.

In total, 44–75% of patients with a Qp/Qs outside the normal range prior to correction were normalized following correction, and no patient with a known shunt was reclassified to normal. This differs from previous findings^17^ with an increase in calculated shunts following baseline correction. That study focused on patients with congenital heart conditions without shunts (*n* = 24), which is in contrast to the current study of patients without known shunts, and a subgroup of verified shunts. Furthermore, they used a static gel phantom^8^ to identify baseline phase offset, and in our current study we used stationary tissue within the acquired image slice. In that study, a free-breathing sequence was also used, and there was a trend of a decreased range of Qp/Qs values following baseline correction, in agreement with the results of the current study. With only 24 participants it is possible that the study suffered from being underpowered. It is also possible that the current study was underpowered, as the normal variation of Qp/Qs in a clinical population without cardiac shunts was unknown at the start of this study, making it difficult to do an *a priori* power calculation. However, given that no patients with verified shunts were reclassified to normal, the current study suggests that stationary tissue background correction can be performed in a clinical setting to reduce measurement errors and increase diagnostic precision. Furthermore, the variations observed in this study could be used as a basis for future studies regarding precision and measurement variability in PC-CMR flow quantification. The aortic flow quantified by phase contrast, was lower compared to the stroke volume quantified by cine, as expected, since the aortic flow is acquired anatomically after the origin of coronary arteries. There was a consistent lower aortic flow using all vendors, however the differences were smaller following stationary tissue background correction in all vendors, suggesting high accuracy of phase contrast flow imaging without quantification tool dependency, and that background correction may reduce inter-method differences.

No patient characteristic affected precision, see Supplemental Material. The lack of statistical significance may be related to the increased statistical demands of repeated testing. Therefore, it would be of interest to perform a more focused analysis in a separate cohort on the parameters that showed potential such as: high BSA, low height, low cardiac output, greater area difference between anterior and posterior image halves, angulations in anterior-posterior, and right to left slice orientation.

There was an increase in precision of Qp/Qs using Vendor 1 and using quadratic correction in Vendor 2, which is supported by several other studies^10,11,18–21^. There was no difference in the variability of the corrected Qp/Qs between the different software programs, and the intra- and inter-observer variability was low. Taken together, these findings suggest that stationary tissue background correction has clinical utility.

Stationary tissue background correction of phase contrast velocity encoded images was integrated in the clinical software products in this study, and the correction takes little additional time to perform. The number of patients with a Qp/Qs outside of the normal range decreased with over 50% following stationary background correction, and no patient with a known cardiac shunt was reclassified to normal. The intra- and inter-observer variability was low across all vendors, in line with previous findings^22,23^. The high reproducibility of flow measurements adds to the clinical utility of flow quantification, and furthermore, high reproducibility is possible to obtain even by less experienced CMR readers^22^. The data suggest that it is of importance to perform stationary tissue background correction in the clinical setting to reduce variability and increase diagnostic precision. Other populations that would be interesting to evaluate stationary tissue background correction in, include patients with valvular disease, healthy volunteers and patients with arrythmias.

One major limitation in this study is that an independent *in vivo* reference method to quantify Qp/Qs is missing in the heterogenous clinical population. The two software products used in this study are the two clinically available at our site, and there are probably more software products for stationary tissue background correction that could be evaluated. Several studies have used a gel phantom as stationary correction for measurement errors in flow quantification^18,21,24^. However, that approach may be more time consuming, thus limiting its clinical adoption. Also, it has recently been shown in a multi-vendor, multi-center approach that stationary tissue background correction reduces phase offset with an efficacy comparable to phantoms^25^. Furthermore, averaging during free-breathing flow imaging in the aorta does not differ from breath-held imaging^26^. However, it is of interest to evaluate free-breathing compared to breath-held techniques in terms of stationary tissue background correction.

In conclusion, stationary tissue background correction reduces the number of patients with Qp/Qs outside the normal range by more than 50%, and does not reclassify patients with verified cardiac shunts to normal. There was no difference in the corrected precision of Qp/Qs between the evaluated software solutions. Stationary tissue background correction may be used in clinical patients to increase diagnostic precision.

## Supplementary information

Supplementary information.
