# Supplementary material for: Stationary tissue background correction increases the precision of clinical evaluation of intra-cardiac shunts by cardiovascular magnetic resonance
Source: Sci Rep. 2020 Mar 19;10:5053. doi: 10.1038/s41598-020-61812-7 (PMC7081189; doi:10.1038/s41598-020-61812-7)
Supplement: Supplementary file 1 — Supplementary information. [file 41598_2020_61812_MOESM1_ESM.docx]

**Supplemental material**

**Stationary tissue background correction increases the precision of clinical evaluation of intra-cardiac shunts by cardiovascular magnetic resonance**

Jannike Nickander^1^, Magnus Lundin^1^, Goran Abdula^1^, Jonas Jenner^1^, Eva Maret^1^, Peder Sörensson^2^, Einar Heiberg^3,4^, Andreas Sigfridsson^1^, Martin Ugander^1,5§^

^1^Department of Clinical Physiology, Karolinska University Hospital, Karolinska Institutet, Stockholm, Sweden

^2^Department of Medicine, Unit of Cardiology, Karolinska Institutet, and Karolinska University Hospital, Stockholm, Sweden

^3^ Department of Clinical Physiology, Lund University, and Skåne University Hospital

^4^ Wallenberg Centre for Molecular Medicine, Lund University, Skåne University Hospital, Sweden

^5^Kolling Institute, Royal North Shore Hospital, and Northern Clinical School, Sydney Medical School, University of Sydney, Sydney, Australia

^§^Corresponding author

Email addresses:

JN: jannike.nickander@ki.se

ML: [mlundin.se@gmail.com](mailto:mlundin.se@gmail.com)

GA: [goran.abdula@sll.se](mailto:goran.abdula@sll.se)

JJ: [jonas.jenner@sll.se](mailto:jonas.jenner@sll.se)

EM: eva.maret@ki.se

PS: peder.sorensson@sll.se

EH: [einar@heiberg.se](mailto:einar@heiberg.se)

AS: andreas.sigfridsson@gmail.com

^§^MU: [martin.ugander@gmail.com](mailto:martin.ugander@gmail.com), Kolling Building, Level 12, Royal North Shore Hospital, St Leonards, NSW 2065, Australia, +46709850088,

**Supplemental Table 1. Patient characteristics and Qp/Qs in Vendor 1.** Significance level was set to *p*=0.0025, and thereby no characteristic increased precision.

| **Patient characteristics Vendor 1** | **Qp/Qs**  **(Median [Inter quartile range])** | **Qp/Qs Corrected (Median [Inter quartile range])** | **p-value** |
| --- | --- | --- | --- |
| **Lower age** | 1.00 [0.94-1.03] | 1.04 [0.98-1.06] | 0.77 |
| **Higher age** | 1.09 [1.01-1.19] | 1.09 [1.04-1.15] | 0.18 |
| **Lower BSA** | 1.00 [0.95-1.08] | 1.04 [0.98-1.10] | 0.39 |
| **Higher BSA** | 1.05 [1.00-1.15] | 1.06 [1.01-1.11] | 0.04 |
| **Lower BMI** | 1.01 [0.96-1.09] | 1.06 [0.99-1.10] | 0.46 |
| **Higher BMI** | 1.05 [0.98-1.14] | 1.06 [1.01-1.12] | 0.09 |
| **Lower height** | 1.02 [0.96-1.09] | 1.06 [1.00-1.11] | 0.17 |
| **Higher height** | 1.02 [0.98-1.14] | 1.06 [1.00-1.11] | 0.15 |
| **Lower weight** | 1.00 [0.96-1.07] | 1.04 [0.98-1.09] | 0.43 |
| **Higher weight** | 1.06 [1.00-1.17] | 1.07 [1.01-1.13] | 0.03 |
| **Lower cardiac output** | 1.03 [0.96-1.14] | 1.06 [1.00-1.12] | 0.02 |
| **Higher cardiac output** | 1.02 [0.98-1.10] | 1.05 [1.00-1.10] | 0.28 |
| **Lower angulation RL** | 1.02 [0.97-1.09] | 1.06 [1.01-1.10] | 0.28 |
| **Higher angulation RL** | 1.03 [0.96-1.14] | 1.06 [1.00-1.11] | 0.07 |
| **Lower angulation AP** | 1.02 [0.96-1.15] | 1.06 [1.00-1.13] | 0.09 |
| **Higher angulation AP** | 1.02 [0.97-1.08] | 1.06 [1.01-1.10] | 0.32 |
| **Lower angulation FH** | 1.03 [0.97-1.09] | 1.06 [0.99-1.10] | 0.32 |
| **Higher angulation FH** | 1.02 [0.97-1.14] | 1.06 [1.00-1.13] | 0.07 |
| **Lower area difference ant-post*** | 1.01 [0.96-1.07] | 1.05 [1.00-1.09] | 0.46 |
| **High area difference ant-post*** | 1.07 [0.98-1.14] | 1.06 [1.00-1.13] | 0.13 |

* Difference in correction surface area between anterior and posterior images halves. AP= anterior-posterior, RL=right-left, FH=feet-head, BSA=body surface area, BMI=body mass index. P-values denote non-parametric Levene’s test.

**Supplemental Table 2. Patient characteristics and Qp/Qs in Vendor 2 with linear correction.** Significance level was set to p=0.0025, and thereby no characteristic increased precision.

| **Patient characteristics Vendor 2 Linear** | **Qp/Qs**  **(Mean ± SD)** | **Qp/Qs Corrected**  **(Mean ± SD)** | | **p-value** |
| --- | --- | --- | --- | --- |
| **Lower age** | 1.00±0.08 | 1.04±0.07 | 0.20 | |
| **Higher age** | 1.08±0.12 | 1.08±0.11 | 0.25 | |
| **Lower BSA** | 1.02±0.10 | 1.07±0.08 | 0.35 | |
| **Higher BSA** | 1.06±0.12 | 1.05±0.10 | 0.70 | |
| **Lower BMI** | 1.02±0.09 | 1.06±0.08 | 0.19 | |
| **Higher BMI** | 1.06±0.12 | 1.06±0.09 | 0.24 | |
| **Lower height** | 1.04±0.12 | 1.07±0.08 | 0.03 | |
| **Higher height** | 1.04±0.10 | 1.05±0.09 | 0.58 | |
| **Lower weight** | 1.02±0.09 | 1.06±0.09 | 0.30 | |
| **Higher weight** | 1.07±0.12 | 1.06±0.10 | 0.24 | |
| **Lower cardiac output** | 1.05±0.11 | 1.08±0.08 | 0.04 | |
| **Higher cardiac output** | 1.03±0.11 | 1.04±0.09 | 0.37 | |
| **Lower angulation RL** | 1.04±0.11 | 1.07±0.10 | 0.53 | |
| **Higher angulation RL** | 1.04±0.11 | 1.05±0.08 | 0.03 | |
| **Lower angulation AP** | 1.05±0.12 | 1.05±1.10 | 0.17 | |
| **Higher angulation AP** | 1.04±0.10 | 1.07±0.08 | 0.15 | |
| **Lower angulation FH** | 1.04±0.10 | 1.07±0.08 | 0.26 | |
| **Higher angulation FH** | 1.05±0.12 | 1.04±0.09 | 0.10 | |
| **Lower area difference ant-post*** | 1.05±0.11 | 1.05±0.10 | 0.33 | |
| **High area difference ant-post*** | 1.03±0.11 | 1.07±0.09 | 0.07 | |

* Difference in correction surface area between anterior and posterior images halves. AP= anterior-posterior, RL=right-left, FH=feet-head, BSA=body surface area, BMI=body mass index. P-values denote F-test.

**Supplemental Table 3. Patient characteristics and Qp/Qs in Vendor 2 with quadratic correction.** Significance level was set to p=0.0025, and thereby no characteristic increased precision.

| **Patient characteristics Vendor 2 Quadratic** | **Qp/Qs**  **(Mean ± SD)** | **Qp/Qs Corrected**  **(Mean ± SD)** | **p-value** |
| --- | --- | --- | --- |
| **Lower age** | 1.00±0.08 | 1.04±0.07 | 0.25 |
| **Higher age** | 1.08±0.12 | 1.04±0.10 | 0.25 |
| **Lower BSA** | 1.02±0.10 | 1.04±0.08 | 0.33 |
| **Higher BSA** | 1.06±0.12 | 1.04±0.09 | 0.07 |
| **Lower BMI** | 1.02±0.09 | 1.04±0.07 | 0.14 |
| **Higher BMI** | 1.06±0.12 | 1.04±0.10 | 0.15 |
| **Lower height** | 1.04±0.12 | 1.04±0.08 | 0.03 |
| **Higher height** | 1.04±0.10 | 1.03±0.09 | 0.39 |
| **Lower weight** | 1.02±0.09 | 1.03±0.08 | 0.49 |
| **Higher weight** | 1.07±0.12 | 1.04±0.09 | 0.07 |
| **Lower cardiac output** | 1.05±0.11 | 1.04±0.08 | 0.05 |
| **Higher cardiac output** | 1.03±0.11 | 1.03±0.09 | 0.23 |
| **Lower angulation RL** | 1.04±0.11 | 1.03±0.09 | 0.27 |
| **Higher angulation RL** | 1.04±0.11 | 1.04±0.08 | 0.04 |
| **Lower angulation AP** | 1.05±0.12 | 1.03±0.09 | 0.03 |
| **Higher angulation AP** | 1.04±0.10 | 1.04±0.08 | 0.42 |
| **Lower angulation FH** | 1.04±0.10 | 1.04±0.09 | 0.33 |
| **Higher angulation FH** | 1.05±0.12 | 1.04±0.09 | 0.04 |
| **Lower area difference ant-post*** | 1.05±0.11 | 1.02±0.10 | 0.23 |
| **Higher area difference ant-post*** | 1.03±0.11 | 1.05±0.09 | 0.03 |

* Difference in correction surface area between anterior and posterior images halves. AP= anterior-posterior, RL=right-left, FH=feet-head, BSA=body surface area, BMI=body mass index. P-values denote F-test.

## Qp/Qs in all patients

Using Vendor 1, linear correction increased Qp/Qs magnitude and precision compared to uncorrected Qp/Qs (median [interquartile range], 1.06 [1.00-1.11] vs 1.02 [0.97-1.12], *p*=0.002, *p*=0.04 for variability). Using Vendor 2, Qp/Qs magnitude and precision remained unchanged following linear correction compared to uncorrected Qp/Qs (1.06±0.09 vs 1.04±0.11, *p*=0.06, *p*=0.06 for variability). Using Vendor 2, Qp/Qs magnitude remained unchanged but precision increased following quadratic correction, (1.04±0.09 vs 1.04±0.11, *p*=0.62, *p*=0.03 for variability), *Figures* 1 and *2.*

## Vendor 1 compared to Vendor 2

Uncorrected Qp/Qs in Vendor 1 had a higher variability compared to Vendor 2, but the magnitude did not differ (1.02 [0.97-1.12] vs 1.03 [0.97-1.10], *p*=0.18, *p*=0.02 for variability). There was no difference in corrected Qp/Qs between Vendor 1 and Vendor 2 with linear correction (1.06±0.09 vs 1.06±0.09, *p*=0.91, *p*=0.80 for variability), nor between Vendor 2 with quadratic correction and Vendor 1 with linear correction (1.04±0.09 vs 1.06±0.09, *p*=0.06, *p*=0.55 for variability).

## Change in aortic and pulmonary flow

Change in individual flow velocities following background correction were calculated as the difference between corrected and uncorrected measurement expressed as percent of their mean. Correction using Vendor 1 resulted in a change in aortic flow of 4.3±4.4%, and a change in pulmonary flow of 6.4±3.9%, *p*<0.001. Using Vendor 2 and linear correction, the change in aortic flow was 5.7±4.9%, and in pulmonary flow was 8.4±4.6%, *p*<0.001. Using Vendor 2 and quadratic correction, the change in aortic flow was 8.7±6.2%, and in pulmonary flow was 9.4±5.1%, *p*=0.29.

## Figures

Figure 1. Change in median (boxplots) and mean (error bars) values for all patients following stationary tissue background correction (n=91). Qp/Qs was higher following linear correction with Vendor 1 compared to uncorrected measurements, whereas Qp/Qs was unchanged following linear and quadratic correction with Vendor 2 compared to uncorrected measurements. *P*-values denote Wilcoxon signed-rank test, or the paired t-test as appropriate.

Figure 2. Differences in precision following linear and quadratic correction with Vendor 1 and Vendor 2 for all patients (n=91). The precision in Qp/Qs increased following linear correction in Vendor 1. Linear correction did not increase precision in Qp/Qs in Vendor 2, however quadratic correction did increase precision in Qp/Qs in Vendor 2. *P*-values denote F-test or non-parametric Levene’s test as appropriate.
